# Sex/Gender as a Factor That Influences Transcranial Magnetic Stimulation Treatment Outcome: Three Potential Biological Explanations

**DOI:** 10.3389/fpsyt.2022.869070

**Published:** 2022-04-29

**Authors:** Colleen A. Hanlon, Daniel M. McCalley

**Affiliations:** ^1^Department of Cancer Biology, Wake Forest School of Medicine, Winston-Salem, NC, United States; ^2^Department of Psychiatry and Behavioral Sciences, Medical University of South Carolina, Charleston, SC, United States

**Keywords:** transcranial magnetic stimulation, craniofacial anatomy, morphology, hormones, sex differences

## Abstract

Repetitive transcranial magnetic stimulation (rTMS) is a non-invasive brain stimulation technique which is now being used in psychiatry clinics across the world as a therapeutic tool for a variety of neural-circuit based disorders (e.g., major depression, obsessive compulsive disorder, substance use disorders, post-traumatic stress disorder, headache, pain). The higher volume of use and publication of multiple large-scale clinical trials has provided researchers with a unique opportunity to retrospectively evaluate factors influencing TMS treatment responses in large samples of patients. While many studies have focused on TMS protocol parameters as moderators of treatment efficacy, sex/gender is another critical, often overlooked factor influencing TMS treatment outcome. Women, especially during periods of high estradiol, appear to be particularly sensitive to the therapeutic effects of rTMS. This manuscript makes a case for three potential biological explanations for these findings. Drawing on literature from cranio-facial anatomy, neuroimaging, and neuroendocrine fields, we posit that observed increases in response rates of women in clinical rTMS trials may be related to: (1) Closer proximity of the brain to the scalp at the prefrontal cortex, leading to larger TMS induced electric fields especially at the medial prefrontal cortex, (2) Greater gray matter density and gyrification in the prefrontal cortex, and (3) High levels of estradiol which facilitate cortical excitability. These biological explanations are empirical ideas which have been evaluated in laboratory studies and lend themselves to prospective evaluation in multisite clinical rTMS trials. The existing literature on this topic and these three potential biological explanations all indicate that the TMS field should routinely evaluate sex/gender (and associated biological metrics like scalp-to-cortex distance, gray matter density, estradiol/progesterone levels) as a factor that may influence treatment outcome.

## Introduction

Repetitive transcranial magnetic stimulation (rTMS) has seen incredible growth as a therapeutic approach to neuropsychiatric disease over the past 20 years. A variety of TMS pulse protocols and TMS device companies have received clearance for therapeutic use in multiple populations (e.g., major depressive disorder, obsessive compulsive disorder, headache, smoking cessation) on multiple continents. There is now widespread therapeutic use of TMS across the globe which has provided researchers a unique opportunity to retrospectively evaluate factors influencing TMS treatment responses in large samples of patients. While many studies have focused on TMS protocol parameters as moderators of treatment efficacy (e.g., frequency, interpulse intervals, sessions, inter session intervals), a recent publication by Sackeim et al. (1) highlights another critical, often overlooked factor influencing TMS treatment outcome–sex/gender.

In this retrospective study, Sackeim et al. (1) evaluated patient outcome data from 5,010 individuals that had received TMS for treatment resistant depression (10 Hz to the left dorsolateral prefrontal cortex). The response and remission rates were comparable to previous studies (Intent to treat sample: 58% response, 28% remission; Completer sample: 83% response, 62% remission). They then evaluated the relative influence of various patient level biometrics on TMS treatment outcome, including: Baseline PHQ-score, age, gender, motor threshold, stimulation intensity, total pulses per session, and the total number of sessions. Among these variables, sex/gender had the most powerful influence on treatment outcome. Specifically, when controlling for various protocol and symptom parameters, females were 1.34 times more likely to respond and 1.37 times more likely to achieve remission from major depression relative to males.

While Sackeim et al. (1) have drawn renewed attention to sex/gender as a significant moderator of rTMS treatment outcome in depression, this phenomenon has been well validated in robust meta-analyses in the depression literature. Notably, in one such meta-analysis performed over a span of 16 years (1997–2013) and 54, sham-controlled research studies, there was a positive, linear relationship between percentage of females enrolled in clinical trials and overall reduction in depression severity (2). Despite consistent results demonstrating the importance of incorporating sex and gender as biological variables of interest when assessing TMS treatment outcome, many influential studies in the field have not reported sex/gender differences in their data (3–5). It is possible that some of these groups do not report on this dimension of treatment outcome due to a lack of statistically significant findings. We argue, however, that previous evidence of improved response rates among women as well as the biological bases for these differences presented here warrant increased emphasis on this factor.

So, what is the biological basis for a higher response rate in females? Three hypotheses:

**(1) Bone-structure differences in males and females influence the “realized” electric field.**

One of the most important biological variables that influence the TMS effects on the brain is the distance from the TMS coil to the cortical target. As Maxwell described in the mid-1800s (6), the strength of an electromagnetic field decays exponentially with distance. The application of this 19th century principle to 21st century therapeutic brain stimulation has been elegantly described by leaders in the TMS field (7). For a given TMS pulse strength (% machine output), cortical regions closer to the scalp (e.g., motor cortex), receive a stronger electric field than brain regions that are farther from a scalp location (e.g., frontal pole). TMS is one of the few therapeutic techniques in psychiatry in which the dosing procedure incorporates patient specific calibrations. Specifically, for each patient, investigators will typically identify the resting motor threshold for the hand or the leg (e.g., the minimum amount of current required to induce motor evoked potentials on 50% of the trials). This dosage will then adjusted based on the specific cortical location that will be stimulated (e.g., often 110–120% when stimulating the prefrontal cortex). This fixed scaling procedure was originally created to account for the known increase in distance from the scalp to the cortex as the coil is moved more anterior and rostral from the motor cortex (e.g., when stimulating the dorsolateral prefrontal cortex or the medial prefrontal cortex).

While these scaling metrics may be acceptable on average, they do not account for known differences in craniofacial anatomy between men and women. This is a well-developed body of literature which has been cultivated and harnessed by multiple domains of science- including archeology (8), medical forensics (9), and craniofacial reconstructive surgery (10). There are several key features of bone structure that can be used to reliably differentiate males and females including the shape of the frontal bone and sinuses, the orientation of the orbits, size of the zygomatic bone, tapering of the mandible, and prominence of the inion (11). From the perspective of delivering TMS to the prefrontal cortex, sex-specific patterns of frontal bone topography are likely important for TMS dosing. In males, the declination angle of the frontal bone (spanning the apex to the nasion) is smaller, leading the forehead to appear more rounded. By contrast, in females, the frontal bone is sharper, leading the forehead to appear flatter (11). The shape of the brain within the skull, however, doesn’t vary significantly between men and women. This leads to a situation wherein the distance from the prefrontal cortex to the external surface of the skull is greater for males than females. This is particularly true in the rostral and medial aspect of the prefrontal cortex- wherein the prominent brow protrusion in males adds to the distance.

Current TMS-dosing strategies use the motor system as the calibration point for treatment. Interestingly, scalp-to-cortex distance and skull morphology at the motor cortex does not differ by sex/gender (12, 13), leading men and women to present equivalent resting motor thresholds on average (14). Uniform corrections applied to overcome the increased scalp-to-cortex distance at the prefrontal cortex (e.g., 110–120% of RMT), then, are likely to have a differential influence on men and women. Specifically, this effect may result in women receiving a higher “realized” electric field at the cortex than men (especially at the frontal pole)–which could account for a larger treatment effect.

**FIGURE 1:**
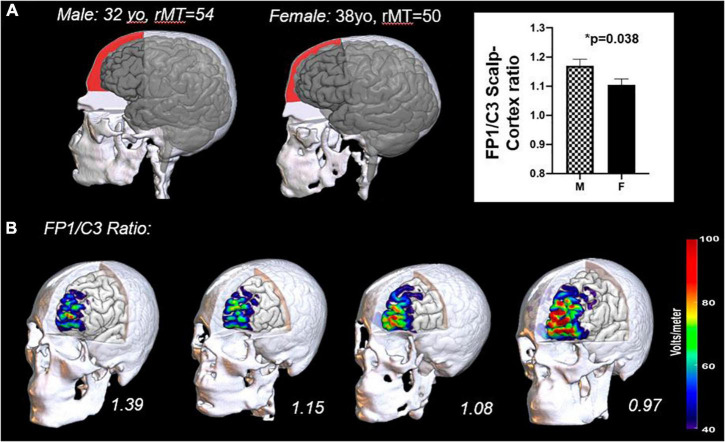
Differences in cranio-facial anatomy in men and women **(A)** and the impact of these anatomical differences on the TMS induced electrical field at the cortical surface **(B)**. The frontal bone of men typically has a lower declination angle (slope) and a more prominent brow ridge extending to the lateral prefrontal cortex than women. This results in a larger distance from the scalp to cortex in men relative to women **(A)**. A representative male and female from a previous study are plotted here with the subdural space from the top of the frontal bone to the level of the nasion shaded (red). Previous data from our laboratory has demonstrated that the scalp-to-cortex distance ratios at the Frontal Pole (FP1) relative to the Motor Cortex (C3) are significantly greater in men (13). The importance of this ratio is underscored by the sensitivity of the TMS induced electric field to small changes in distance **(B)**. Here we have rendered the TMS induced electric field (110% resting motor threshold) for four individuals in the study–two males (left) and two females (right). In as much as the motor threshold continues to be used as a dosing metric for other cortical areas, it would be prudent to incorporate these ratios into dosing protocols.

In our own work, in an analysis of 88 individuals (male, *n* = 58; female, *n* = 30), we analyzed scalp-to-cortex distance and resulting electrical field strength at various stimulation targets for TMS in a sample of healthy controls and Alcohol Use Disorder patients (FP1: left frontal pole, F3: left dorsolateral prefrontal cortex; C3: left motor cortex). Sex/gender consistently emerged as a significant covariate. Women in both groups had a significantly shorter scalp-to-cortex distance than men at the frontal pole (FP1; female: 15.88 ± 2.8 mm; male: 17.79 ± 3.18 mm; *t*_87_ = 2.938, *p* = 0.004, Hedge’s *g* = 0.62) but not at the primary motor cortex (C3; female: 14.02 ± 1.83 mm; male: 14.75 ± 2.52 mm; *t*_87_ = 1.49, *p* = 0.138, Hedge’s *g* = 0.33). Given that TMS is routinely delivered to the prefrontal cortex based on a dose determined at the motor cortex, the smaller ratio of FP1/C3 distance in women relative to men led to a TMS electrical field that was, on average 5–7 V/m stronger at the same stimulation site (13). An example of this is illustrated in Figure 1. Notably, the scalp-to-cortex distance at F3 (left DLPFC) was also shorter in women than men, but the effect size was smaller than the frontal pole (F3; female: 14.49 ± 2.26 mm; male: 15.04 ± 2.50 mm; *t*_87_ = 1.054, *p* = 0.289, Hedge’s *g* = 0.23). [Note: AUD individuals did not have a significantly reduced scalp-to-cortex distance at any tested cortical location. Men and women from both groups were therefore aggregated for this analysis].

These data suggest that craniofacial differences in bone structure lead, on average, to a higher electric field strength at the prefrontal cortex (when the TMS dose is determined based on the motor threshold). This may account for a higher response rate in women–especially in TMS protocols targeting the medial prefrontal cortex. It is not clear, however, that distance alone would account for the observed sex/gender differences when rTMS is delivered to the DLPFC or the motor cortex.

**(2) Gray and white matter differences.**

Another explanation may be differences in gray and white matter integrity in men and women. Previous work has demonstrated that the ability of TMS to propagate to downstream targets (likely a key factor in improving treatment outcome) is dependent on three features of brain structure: (1) Scalp-to-cortex distance (discussed above), (2) Gray matter volume (GMV) at the stimulation site, and (3) White matter fractional anisotropy (FA) (15–17). Specifically, shorter scalp-to-cortex distances, greater GMV and greater FA were associated with elevated TMS-evoked activity in subcortical regions.

There are also known sex/gender differences in gray and white matter among healthy controls that may influence TMS response. After correcting for differences in total intracranial volume, women have greater tissue volume within the frontal and parietal cortices, relative to men (18–20). These increases have regional specificity, with differences occurring in common TMS targets (e.g., medial prefrontal, dorsolateral prefrontal cortex). Women also have a higher gyrification index–a measurement of cortical folding–in the frontal and parietal cortices leading to an increase in gyral surface area (21). Given that the strength of TMS is most intense at the gyral crown perpendicular to the TMS coil (22), this may indicate that electrical fields (1) cover more surface area and (2) more readily propagate to downstream targets in women than men.

There are also sex/gender differences in white matter microstructure, wherein women have lower fractional anisotropy values throughout the brain, relative to men (23–26). Few, however, have suggested that women have regionally specific elevations in fractional anisotropy (27). It is possible then, that broad reductions in white matter fractional anisotropy may attenuate the benefits conferred by the favorable profile of gray matter volume among women relative to men. It will be important for the field of brain stimulation moving forward to consider how these competing influences may impact treatment outcome.

Taken together, both aforementioned hypotheses–craniofacial anatomy and brain tissue volume–suggest that women would likely have a higher brain response to an equivalent TMS stimulation intensity than men, especially when TMS is delivered to the prefrontal cortex. This is consistent with the aforementioned meta-analysis of 54 randomized, sham controlled studies of rTMS for Major Depressive Disorder (MDD) (2). These findings, however, are not universal. One of the first randomized, sham-controlled clinical trials evaluating factors that influence response to rTMS in MDD patients, for example, failed to find a difference in 4-week response rate between men and women [301 individuals from 23 sites, 55% female (28)].

It is therefore unlikely that the first two hypotheses alone dictate gender differences in TMS treatment response. So what, then, is contributing to the variance in these well powered studies?

**(3) Effects of estrogen and progesterone on cortical excitability.**

The variance in observed data may be related to the impact that fluctuations in estradiol and progesterone have on cortical excitability (29–31). Briefly, in women of child-bearing age, the menstrual cycle is often divided into 3 predictable phases: menses, follicular, luteal; and guided by two predominant hormones: estradiol and progesterone. During menses, both hormones are low. During the follicular phase, estradiol rises. During the luteal phase, both estradiol and progesterone are high (32). An extensive amount of clinical and preclinical literature has demonstrated phase-dependent effects on cognition, behavioral flexibility, emotion regulation, and reward sensitivity. Although a review of this literature is beyond the scope of this manuscript, one theme that emerges is that higher levels of estradiol (late follicular phase) are associated with enhanced performance on cognitive tasks, that are not present when progesterone rises (luteal phase). Preclinical studies have demonstrated that estradiol facilitates cortical excitability, likely through glutamatergic mechanisms (33), while progesterone metabolites dampen cortical excitability (34), likely through gamma-aminobutyric acid (GABA).

In a series of two papers Smith et al. tested this hypothesis using TMS (29, 30). In both studies they evaluated cortical excitatory tone and inhibitory tone though paired pulse techniques with interpulse intervals from 2 to 10 ms. This large spectrum of interpulse intervals allowed them to evaluate both paired pulse facilitation (glutamate mediated) and paired pulse inhibition (GABA mediated). Cortical excitability was evaluated when the women were in the early follicular phase, late follicular phase, and the luteal phase. Cortical facilitation was highest when estradiol was high and progesterone was low (late follicular) (30). Cortical facilitation was the lowest when estradiol and progesterone were both high (luteal). These complementary effects of estradiol and progesterone were quickly corroborated by several other groups (31, 35). Importantly, the attenuated excitability in the luteal phase (likely progesterone mediated) was comparable in magnitude to the effect of GABA agonists and glutamate antagonists on cortical excitability (36, 37). This momentum in the motor physiology field, however, did not carry over to the psychiatric research field. In one of the few studies to evaluate the effect of hormone levels on TMS to the DLPFC, Chung et al. (38) demonstrated that in periods of high estrogen, a single session of 10 Hz DLPFC TMS resulted in higher TMS-induced excitability (measured by EEG) than low estrogen or in men (39). There is still a lot of research to be done, however, as the contributions of estradiol and progesterone to potential therapeutic effects of rTMS have not been widely investigated.

Assuming that the effects of these hormones on motor cortex excitability generalize to the prefrontal cortex, dynamic levels of estradiol and progesterone within and between women across the lifecycle may be a significant source of within and between subject variability. In practice, a traditional clinical course of TMS typically last longer than a 28 day menstrual cycle, which would mean that data would like be collected from a single subject across the full spectrum of estradiol and progesterone fluctuation. Moving forward, however, there is momentum toward accelerated TMS design, wherein a full course of TMS (30–36 sessions) is delivered to patients with depression in as little as only 3–5 days (40, 41). For these accelerated TMS designs, it stands to reason that the effects of TMS might be magnified if delivered to women during periods of high estrogen and low progesterone. Women taking various forms of estrogen supplementation (e.g., high estrogen birth control pills, hormone replacement therapy) may also have greater TMS-induced effects on the brain and behavior than individuals with fluctuating hormone levels or post-menopausal women. Post-menopausal women may need a higher dose of TMS (e.g., intensity/session, number of sessions required to produce cortical change) than they would have earlier in life (independent of any age-related changes in brain volume).

To the best of our knowledge, there have been no rigorous TMS studies to investigate the influence of testosterone (which can be converted to estradiol in men and women *via* aromatase) on cortical excitability. Future research may consider a more comprehensive approach to assessing the relative contributions of estradiol, progesterone, and testosterone on cortical excitability and treatment outcome in both men and women.

## Discussion

There is now a large body of research suggesting that sex/gender may be an important factor involved in TMS treatment response. While the majority of the studies evaluating factors that influence therapeutic outcome focus on aspects of the TMS dose and measures of disease severity, there is strong reason to believe that sex/gender may be a potent and transdiagnostic biomarker influencing the effects of TMS on the brain and behavior. Sex/gender is further a factor that applies to healthy controls and patient population.

We have chosen to group the words sex and gender in this perspective for a variety of reasons. Gender is typically used to describe the characteristics of women and men that are socially constructed, while sex is typically used to describe characteristics that are biologically determined (42). Most clinical trials do not specify if the recorded data was biologic sex or self-reported gender. The three potential explanations discussed above, however, are all biological in nature and more tightly related to sex. So, while the higher response rates observed in women in clinical trials may refer to sex or gender, the three potential explanations put forth here are all related to sex as a biological measure. Additionally, it is important to note that the hormones cited in point #3 are present in both sexes, and should probably be considered on a continuum rather than limiting the impact to individuals that are male or female.

## Practical Suggestions Moving Forward

This manuscript highlights the importance of sex/gender as a meaningful biologic variable in therapeutic brain stimulation. There are fundamental differences in craniofacial anatomy and brain tissue density in men and women, which may lead to a larger “realized” TMS dose in women if scalp-to-cortex distance is not considered. We recommend that future studies recruit an even gender distribution in to all arms and take the scalp-to-cortex distance at the target location into account when tailoring the treatment to the individual. While the TMS induced electric field can be modeled with existing software, the field would be well-served by software that is more broadly accessible to individuals without extensive computer programming experience.

Furthermore, naturally fluctuating levels of estradiol and progesterone may also influence treatment outcomes. From a clinical perspective, these data would suggest that women in periods of high estrogen and low progesterone are likely to have the largest neuroplastic response to TMS–a feature that clinicians should consider, especially during accelerated theta burst protocols wherein the full stimulation course is delivered within a few days. Moving forward, clinical TMS trials should systematically evaluate sex/gender as a contribution to the main effects of the study and report the results of the analysis regardless of statistical significance. We recommend future investment by government funding agencies into prospective evaluation of estradiol, progesterone, and testosterone on cortical excitability and therapeutic rTMS outcomes.

## Data Availability Statement

The original contributions presented in the study are included in the article/supplementary material, further inquiries can be directed to the corresponding author.

## Author Contributions

Both authors contributed to all the aspects of this report including conceptualization, writing, and revising, contributed to the article, and approved the submitted version.

## Conflict of Interest

The authors declare that the research was conducted in the absence of any commercial or financial relationships that could be construed as a potential conflict of interest.

## Publisher’s Note

All claims expressed in this article are solely those of the authors and do not necessarily represent those of their affiliated organizations, or those of the publisher, the editors and the reviewers. Any product that may be evaluated in this article, or claim that may be made by its manufacturer, is not guaranteed or endorsed by the publisher.
